# Impact of nirsevimab prophylaxis on paediatric respiratory syncytial virus (RSV)-related hospitalisations during the initial 2023/24 season in Luxembourg

**DOI:** 10.2807/1560-7917.ES.2024.29.4.2400033

**Published:** 2024-01-25

**Authors:** Corinna Ernst, Dritan Bejko, Leo Gaasch, Emilie Hannelas, Isaline Kahn, Charlotte Pierron, Nesrine Del Lero, Claude Schalbar, Elsa Do Carmo, Michel Kohnen, Emmanuelle Andlauer, Pauline Hublart, Silvana Masi, Isabel de la Fuente Garcia, Anne Vergison, Joël Mossong

**Affiliations:** 1 Health Directorate, Luxembourg; 2 ECDC Fellowship Programme, Field Epidemiology path (EPIET), European Centre for Disease Prevention and Control (ECDC), Stockholm, Sweden; 3 National Center for Pediatrics, Centre Hospitalier de Luxembourg, Luxembourg; 4 Microbiology department, Centre Hospitalier de Luxembourg, Luxembourg; 5 Hospital Pharmacy department, Centre Hospitalier de Luxembourg, Luxembourg

**Keywords:** Respiratory Syncytial Virus, Nirsevimab, Real-world evidence, Paediatric hospitalization, Immunisation, Luxembourg

## Abstract

After Luxembourg introduced nirsevimab immunisation against respiratory syncytial virus (RSV), estimated neonatal coverage was 84% (1,277 doses/1,524 births) in 2023. That year, paediatric RSV-related hospitalisations, especially concerning infants < 6 months old (n = 72) seemed to decrease compared to the same period in 2022 (n = 232). In 2023, hospitalised children’s mean age increased (14.4 months vs 7.8 months in 2022; p < 0.001) and hospital-stay length decreased (3.2 days vs 5.1 days; p < 0.001). In infants < 6 months old, intensive-care unit admissions appeared to drop (n = 28 vs 9). This suggests that nirsevimab prophylaxis reduced severe RSV infections, particularly in infants < 6 months old, thereby alleviating healthcare strain.

Respiratory syncytial virus (RSV) infections are a major cause of hospitalisation in young children [1-3]. Because nirsevimab, a novel long-acting monoclonal antibody, demonstrated substantial efficacy in clinical trials, reducing both hospitalisation and disease severity in infants [4,5], the European Medicines Agency (EMA) authorised nirsevimab in the European Union/European Economic Area in October 2022 [6]. Nevertheless, few European countries started implementing immunisation campaigns before the beginning of the 2023/24 RSV season and shortages were moreover reported by the United States Centers for Disease Control and Prevention due to limited availability of the antibody [7]. In October 2023, passive immunisation with nirsevimab was started simultaneously across maternities of all of Luxembourg’s four hospitals [8]. One of our objectives was to estimate the neonate coverage of nirsevimab immunisation in Luxembourg up to mid-December 2023. We also aimed to investigate the effect of this immunisation on children under 5 years of age, by comparing RSV-related paediatric hospitalisation data between weeks 39–52 of 2022 (pre-immunisation period) and of 2023 (post-immunisation) respectively, at Luxembourg's national paediatric hospital.

## Immunisation recommendations, roll-out and calculating coverage

After a surge in RSV cases and related hospitalisations in 2022, RSV became a laboratory-notifiable infection in Luxembourg in early 2023 [9]. In July of that year, Luxembourg's infectious diseases advisory group recommended one-dose nirsevimab prophylaxis for: (i) all neonates born between 1 October 2023 and 30 March 2024; (ii) infants born from 1 January until 30 September 2023 (i.e. catch-up immunisation); and (iii) children under the age of 2 years with risk factors for severe respiratory infection [8]. This recommendation was followed in October 2023 by a national immunisation campaign.

Data on nirsevimab administration in hospitals are regularly collected by the Health Directorate to follow the consumption and available stock. The coverage is calculated on a weekly basis by dividing the number of nirsevimab doses used at the maternity and neonatology departments of the hospitals with the number of births on the same period. Based on the number of nirsevimab doses administered among neonates in all maternity wards across the four Luxembourg hospitals, we calculated the national immunisation coverage from the beginning of October to mid-December 2023.

## Investigating changes between the 2022/23 and 2023/24 epidemics

We conducted a preliminary analysis of RSV-related hospital admissions at Luxembourg's national paediatric hospital – which represents 86% of the total paediatric beds, including all five intensive care beds available in the country.

During the RSV season (weeks 39 to 13), children presenting with typical respiratory symptoms at the hospital are routinely screened for RSV by ID NOW RSV molecular point-of-care test (Abbot Diagnostics, Scarborough, ME, US). Infants under 6 weeks of age testing RSV positive are frequently hospitalised for at least one night as a precautionary measure to closely monitor disease progression. Older infants (> 6 weeks of age) are hospitalised based on their clinical condition (i.e. respiratory parameters, feeding capacities).

### Case definition

An RSV-related case was defined as a hospitalised child below 5 years of age who was screened and tested positive for RSV by molecular methods, upon admission to the paediatric hospital during the early RSV seasons (weeks 39 to 52) of 2022 and 2023.

### Comparison between 2022/23 and 2023/24 respiratory syncytial virus seasons

The hospitalisation data of children infected with RSV were used to describe the 2022/23 (i.e. week 39 2022 to week 13 2023) RSV epidemic and the beginning of the 2023/24 RSV epidemic (i.e. week 39 to 52 2023). Comparisons between some of the cases’ features in weeks 39 to 52 of 2022 and of 2023 respectively, were conducted. These included the age structures of cases in each early RSV season. Moreover, for 2023, the proportions of cases who had not received nirsevimab were also calculated (among overall cases, and among cases up to 6 months old).

We studied changes relating to disease severity from one RSV season to the other, by investigating the cases’ length of stay in hospital, as well as the admissions to paediatric intensive care units (PICU), both for cases overall, and for cases up to 6 months old. We also assessed the need for oxygen support therapy by immunisation status in 2023.

Pearson’s chi-squared test was used to compare categorical variables and t-test for continuous variables. Mann–Whitney U test was used to confirm the analysis for continuous variables. P-values ≤ 0.05 were considered significant. IBM SPSS Statistics (Version 27, Armonk, NY, US) was used for the analysis.

## Result of neonatal passive immunisation coverage estimation

Neonatal coverage in maternity wards from the beginning of October to mid-December 2023 was estimated at 84% (1,277 doses for 1,524 births) ranging from 66% to 94% between maternity wards. Coverage in outpatient settings could not be monitored as there is no immunisation registry. No adverse events associated with the immunisation have been reported to date.

## Description of 2022/23 and 2023/24 epidemics

**Figure 1:**
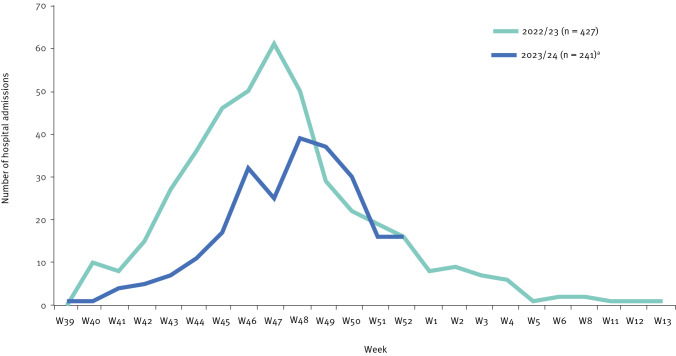
Respiratory syncytial virus (RSV) hospital admission of children under 5 years of age by week in Luxembourg’s national paediatric hospital, Luxembourg, 2022–2023 (n = 668)

In 2023, 241 children under 5 years of age were hospitalised with a laboratory-confirmed RSV infection (i.e. cases), compared with 389 cases in 2022, representing decreases of 38% (389 vs 241) in cases under 5 years of age and 69% (232 vs 72) in cases of infants under 6 months old (Figure 1). The peaks of the respective RSV epidemics occurred in week 47 in 2022 and in week 48 in 2023.

W: week.

^a^ In the figure, the 2023/24 epidemic is only depicted up to week 52.

## Age structure of cases and proportion of cases immunised 

**Figure 2:**
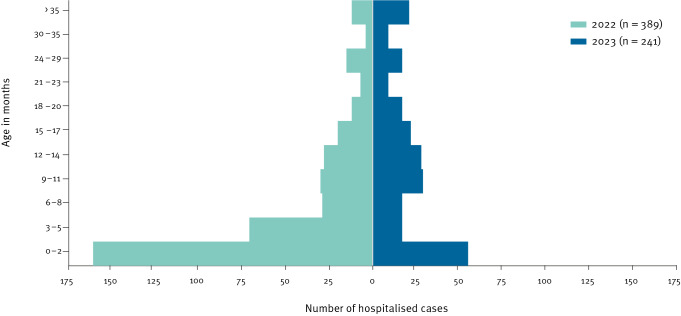
Age distribution of children hospitalised with respiratory syncytial virus (RSV) infection in Luxembourg’s national paediatric hospital in weeks 39–52, Luxembourg, 2022–2023 (n = 630)

During the study periods (weeks 39–52), the mean age of children was significantly higher in 2023 (14.4 months; standard deviation (SD): 12.9) than in 2022 (7.8 months; SD: 10.1; p < 0.001). Infants up to the age of 6 months represented the largest age group of admissions in 2022 (59.6%; 232/389), whereas they accounted for 29.9% (72/241) of admissions (p < 0.001) in 2023 (Figure 2).

Among the 241 children under 5 years of age hospitalised with an RSV infection in 2023, 213 (88.4%) were not immunised with nirsevimab. Among 72 hospitalised infants up to 6 months of age, 47 (65.3%) were not immunised.

## Severity of disease 

The length of hospital stay was significantly reduced from a mean of 5.1 days (SD: 5.4) in 2022 compared to 3.2 days (SD: 2.5) in 2023 (p < 0.001). This reduction was most marked among infants < 6 months old (5.6 days in 2022 vs 3.4 days in 2023, p < 0.001) compared to children ≥ 12 months old (4.2 days in 2022 vs 3.2 days in 2023, p = 0.17), who were not the main target of the immunisation campaign. The total number of RSV-related hospitalisation days decreased from 1,984 in 2022 to 771 in 2023 (p < 0.001).

In 2023, the majority (22/28) of infants up to 6 months of age needing oxygen supplementation were not immunised. Nine of 13 infants who received Optiflow nasal high flow therapy, and six of nine infants admitted to PICU were not immunised.

The proportion of hospitalised children aged less than 5 years admitted to PICU decreased from 9.3% (36/389) in 2022 to 6.2% (15/241) in 2023 (p = 0.16). In infants up to 6 months of age, we observed an overall reduction of hospital (232 to 72) and PICU (28 to 9) admissions.

## Discussion

In clinical trials nirsevimab showed between 74% and 86% efficacy against medically-attended lower respiratory tract infections caused by RSV in healthy infants [4]. In a context of moderate to high immunisation coverage (84%) among neonates, our study provides early real-world evidence of nirsevimab immunisation protecting infants from severe RSV disease in Luxembourg. We hope for a further increase in coverage in infants in the future.

An early onset and challenging RSV season in Luxembourg occurred in 2022, marked by a surge in paediatric admissions and hospital capacity limitations, including transfer of PICU patients to other units abroad.

Most striking and most likely attributable to nirsevimab immunisation was a different age structure between the 2023/24 and 2022/23 RSV seasons, particularly among the most vulnerable, i.e. infants under 6 months of age. The increased average age of admitted children and the reduced number of infants requiring hospitalisation may be attributed to an effect of the administration strategy shortly after birth. Most hospitalised children had not yet received a nirsevimab immunisation, supporting results from clinical studies on the protective efficacy of nirsevimab against medically-attended RSV-associated lower respiratory tract infection [5,10].

In 2023, we observed a reduced number of hospital and PICU admissions among infants with RSV infection, while screening and admission criteria did not change. This positive development contributed to the maintenance of routine hospital planning, allowing most scheduled medical interventions to proceed as planned, in contrast to the previous year.

We are aware of and acknowledge the following limitations in this descriptive study. First, we compared only two consecutive seasons, and it would be important to investigate a longer time span. The high intensity of the 2022 epidemic could be partially attributed to immunity depletion resulting from reduced RSV circulation during the implementation of COVID-19 mitigation measures [11]. Second, our study did not cover the full 2023/24 RSV season, although the peak had already occurred. Finally, we were unable to estimate the prophylactic coverage in infants immunised in outpatient settings, i.e. those mainly born before October 2023. However, we consider that our small country observation is important to encourage other countries to evaluate this intervention on a larger scale.

## Conclusion

Our study shows the impact of nirsevimab in mitigating severe RSV disease among infants during the first RSV season following the national implementation of passive immunisation achieving high coverage in Luxembourg. There was a significant increase in age of hospitalised children, and most severe RSV-related hospitalisations occurred in non-immunised children. The reduction of RSV-related ward occupancy contributed to hospital resources not being overwhelmed.
